# Establishing the relevance of endocrine‐disrupting effects for nontarget vertebrate populations

**DOI:** 10.1002/ieam.4116

**Published:** 2019-02-26

**Authors:** Mark Crane, Nina Hallmark, Laurent Lagadic, Katharina Ott, Dan Pickford, Thomas Preuss, Helen Thompson, Pernille Thorbek, Lennart Weltje, James R Wheeler

**Affiliations:** ^1^ AG‐HERA Faringdon United Kingdom; ^2^ Bayer SAS Crop Science Division Regulatory Toxicology Sophia‐Antipolis Cedex France; ^3^ Bayer AG Crop Science Division Environmental Safety Monheim am Rhein Germany; ^4^ BASF SE Crop Protection – Ecotoxicology Limburgerhof Germany; ^5^ Syngenta Ltd Jealott's Hill International Research Station Bracknell United Kingdom; ^6^ Current address: BASF SE APD/EE Limburgerhof Germany; ^7^ Corteva Agriscience Agriculture Division of DowDuPont Park Abingdon Oxfordshire United Kingdom

European Commission (EC) criteria to identify the endocrine‐disrupting properties of plant protection products (PPPs) have been developed in a regulation for application across the European Union (EU) beginning 10 November 2018 (EC 2018), with implementation of this regulation supported by an accompanying guidance document (ECHA/EFSA 2018).

The endocrine assessment required for PPPs under this regulation is hazard based rather than risk based, but there are inherent challenges in such hazard‐based regulations (Nordlander et al. 2010). For example, 1) safe products may be restricted (because humans or animals may never be exposed to doses that cause adverse effects), 2) beneficial products may be restricted with consequent loss of consumer benefits, 3) restrictions on the use of one product may lead to increased use of another product with equal or greater risks, and 4) product stigmatization and loss of incentives to innovate may occur.

Based on a review of the literature and interviews with regulators, academics, policymakers, politicians, and other stakeholders across the EU, Lofstedt (2011) concluded that political pressure to regulate on the basis of hazard or risk varied among EU Member States, and inconsistent positions were taken by Member States depending upon whether hazard assessment supported or threatened their respective national interests. Lofstedt (2011) found that Europe‐wide regulation based on weaker scientific approaches, such as hazard‐ instead of risk‐based assessment, has been facilitated by several factors, including

- the rise of the “post‐trust society,”
- a lack of interest in environmental regulatory issues on the political center right,
- an increase in vote‐winning “game‐playing” by politicians in policy areas that have only limited impacts on their own electorates,
- different cultural values in different Member States,
- a lack of effective communication and agreement among different national and European regulatory agencies,
- product stigmatization, and
- increasingly negative public perceptions of chemicals.

In addition to these general weaknesses of a hazard‐based approach, further weaknesses are apparent in the ECHA/EFSA (2018) guidance for the identification of endocrine disruptors for PPPs due to inconsistencies between what the regulation requires and what the guidance recommends. Inconsistency is especially evident in guidance concerning vertebrate wildlife population relevance, as shown by the following 2 direct quotes:

1. “When assembling and assessing the line of evidence, any available epidemiological studies should be considered as supportive evidence for the evaluation of whether an [endocrine disruptor] ED is likely to have adverse effects for humans. However, they cannot be used to override or dismiss evidence of adversity found in laboratory studies, nor can they replace laboratory studies. Similarly, when assembling the lines of evidence for non‐target organisms any field and monitoring studies and population modelling can be considered as supportive evidence” (ECHA/EFSA 2018, p 20).
2. “Effects on growth, development, [and] reproduction in single species are generally regarded relevant for the maintenance of the wild population. Therefore, the relevance of such effects at the population level should be assumed when determining the adversity in the absence of appropriate scientific data demonstrating non‐relevance” (ECHA/EFSA 2018, p 22).

These statements contradict the PPP regulation clauses that a substance should be considered to have endocrine‐disrupting properties “unless there is evidence demonstrating that the adverse effects identified are not relevant at the (sub)population level for non‐target organisms,” and that “adequate, reliable and representative field or monitoring data and/or results from population models shall as well be considered where available” [EC 2018].

This inconsistency between regulation and guidance threatens to make it difficult to disprove the relevance of endocrine effects on nontarget wildlife vertebrate populations because of an apparent reliance on a hazard assessment approach in which any indication of an endocrine‐mediated individual adverse effect in any laboratory test is considered to indicate that a PPP is an endocrine disruptor with wildlife population relevance. This excludes any consideration of population relevance based on modeling or field data, which is clearly not the intention of the regulation.

Participants at a recent SETAC Pellston Workshop^®^ on Endocrine‐Active Substances (Matthiessen et al. 2017) suggested that regulation of endocrine‐disrupting chemicals based on hazard alone may be partly driven by a perception that there is no toxic threshold for these substances. The workshop concluded that 1) it is impossible to prove that toxic thresholds do not exist, 2) a viable physiological basis for no thresholds has not been clearly identified, and 3) there is no evidence that no‐threshold effects apply to populations. Workshop participants determined that thresholds of toxicity existed for all 6 data‐rich case study substances examined, and that theoretical considerations suggested that endocrine systems could not actually function if such thresholds were absent.

Agreement on scientifically defensible methods to assess adverse population effects of PPPs in the context of endocrine‐disrupting activity is urgently required. In an associated paper (Crane et al. this issue), we propose a practical and scientifically robust way to bridge the gap between the guidance and the legal requirements of the regulation. The approach maintains the hazard‐based intent of the PPPs regulation and introduces the necessary steps between demonstration of individual adverse endocrine effects in laboratory organisms and demonstration of adverse effects on wildlife populations. We hope that this proposal initiates a debate among relevant stakeholders to establish scientifically sound regulatory solutions to resolve the inconsistency between the legally mandated regulation (EC 2018), the supporting guidance document (ECHA/EFSA 2018), and the intent of science‐based regulation in the EU.
